# Epidemiologic and clinical updates on viral infections in Saudi Arabia

**DOI:** 10.1016/j.jsps.2024.102126

**Published:** 2024-06-08

**Authors:** Noura M. Alshiban, Munirah S. Aleyiydi, Majed S. Nassar, Nada K. Alhumaid, Thamer A. Almangour, Yahya M.K. Tawfik, Laila A. Damiati, Abdulaziz S. Almutairi, Essam A. Tawfik

**Affiliations:** a Advanced Diagnostics and Therapeutics Institute, Health Sector, King Abdulaziz City for Science and Technology, Riyadh 11442, Saudi Arabia; b Department of Clinical Pharmacy, College of Pharmacy, King Saud University, Riyadh 11451, Saudi Arabia; c Department of Biological Sciences, College of Science, University of Jeddah, Jeddah 23218, Saudi Arabia; d Field Epidemiology Program (FETP), Ministry of Health, Riyadh 12631, Saudi Arabia

**Keywords:** Viruses, Viral infections, Prevention, Vaccines, Saudi Arabia

## Abstract

In the past two decades, the world has witnessed devastating pandemics affecting the global healthcare infrastructure and disrupting society and the economy worldwide. Among all pathogens, viruses play a critical role that is associated with outbreaks due to their wide range of species, involvement of animal hosts, easily transmitted to humans, and increased rates of infectivity. Viral disease outbreaks threaten public health globally due to the challenges associated with controlling and eradicating them. Implementing effective viral disease control programs starts with ongoing surveillance data collection and analyses to detect infectious disease trends and patterns, which is critical for maintaining public health. Viral disease control strategies include improved hygiene and sanitation facilities, eliminating arthropod vectors, vaccinations, and quarantine. The Saudi Ministry of Health (MOH) and the Public Health Authority (also known as Weqayah) in Saudi Arabia are responsible for public health surveillance to control and prevent infectious diseases. The notifiable viral diseases based on the Saudi MOH include hepatitis diseases, viral hemorrhagic fevers, respiratory viral diseases, exanthematous viral diseases, neurological viral diseases, and conjunctivitis. Monitoring trends and detecting changes in these viral diseases is essential to provide proper interventions, evaluate the established prevention programs, and develop better prevention strategies. Therefore, this review aims to highlight the epidemiological updates of the recently reported viral infections in Saudi Arabia and to provide insights into the recent clinical treatment and prevention strategies.

## 1 Introduction

The world has recently witnessed a wave of emerging and reemerging viral infections such as dengue fever, Coronavirus disease 2019 and influenzas A. Emerging infectious diseases occur when the contagious pathogen is introduced to the host population, leading to an increased incidence rate in humans and threatening future disease outbreaks (McArthur, 2019). Viral infection pandemics are a global health concern affecting the global healthcare infrastructure and disrupting society and the economy worldwide (Bhadoria et al., 2021). Devastating viral pandemics in the past two decades have primarily been caused by respiratory viral infections like coronaviruses causing severe acute respiratory syndrome (SARS) outbreak in 2002, Middle Eastern respiratory syndrome (MERS) outbreak in 2012, and the Coronavirus disease 2019 (SARS-CoV-2) pandemic in 2019. Also, influenza viruses are among the respiratory viral pathogens causing the Swine flu (influenza A H1N1) pandemic in 2009. Vector-borne viruses were also spread like the Ebola virus disease outbreak in 2014, and the Zika virus disease outbreak in 2015. It is evident that among all pathogens, viruses play a significant role in the association of disease outbreaks due to their wide range of species, involvement of animal hosts, easily transmitted to humans, and increased rates of infectivity (Bhadoria et al., 2021, Baker et al., 2022).

The challenges of controlling viral diseases raise a global dilemma. The vast variation of viruses' epidemiology and mechanism of pathogenicity makes it challenging to control and eradicate viral infections (Goldenthal et al., 1996). Viral disease management strategies vary depending on mortality rates, the severity of infections, transmissibility, and transmission patterns (e.g., airborne transmission and zoonosis). The management strategies for controlling viral diseases include proper hygiene, improved sanitation, eliminating arthropod vectors, vaccinations, and quarantine (MacLachlan and Dubovi, 2017). The advancement of medicines, easy access to health care, and improved hygiene and sanitation facilities have reduced the burden of infectious diseases, primarily respiratory and diarrheal infectious diseases. Furthermore, modern science has proven its efficacy in combating emerging viral disease threats with the prompt development of vaccines similar to what was witnessed in the SARS-COV-2 era (Baker et al., 2022). Nevertheless, all these advancements would not have been possible without an effective disease control program. Implementing viral disease control measures is highly dependent on surveillance systems. Systematic and regular surveillance data collection and analyses to detect infectious disease trends and patterns provide helpful information for developing disease prevention strategies (MacLachlan and Dubovi, 2017).

The Saudi MOH and the Public Health Authority are responsible for public health surveillance to control and prevent viral diseases. The viral diseases that are reported to the MOH can be categorized into Hepatitis diseases, viral hemorrhagic fevers (VHFs), respiratory viral diseases, exanthematous viral diseases, neurological viral diseases and viral conjunctivitis (Assiri and AlSwaidi, 2017). Monitoring trends and detecting changes in these viral diseases is essential to provide proper interventions, evaluate the established prevention programs, and develop better prevention strategies. Therefore, this review focuses on the epidemiological considerations of the recently reported viral infections in Saudi Arabia and provides insights into their treatment and prevention strategies.

## 2 Hepatitis

**Fig. 1:**
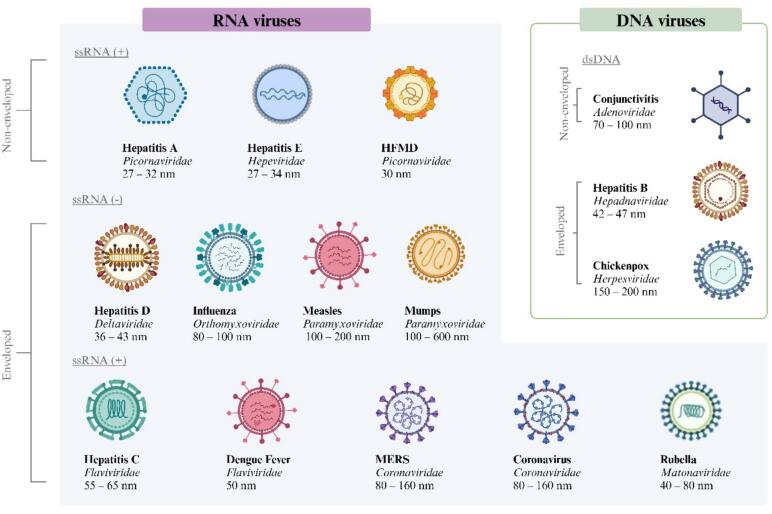
Represents the classification of viruses based on the nucleic genome, which is (DNA) or (RNA; whether positive sense or negative sense). Also, it can be called enveloped or nonenveloped according to the morphological features. Human viruses belong to different families with diameter variations.

Viral hepatitis, which includes several different structures (Fig. 1), remains to be a global public health concern. According to the World Health Organization (WHO), it is estimated that 354 million people worldwide live with hepatitis B or C and 1.5 million people are newly infected with chronic hepatitis B and C (“CDC,” 2018). Updated prevalence and estimate studies of the different types of viral hepatitis in Saudi Arabia are lacking, though, according to the Coalition for Global Hepatitis Elimination in 2019, the prevalence of chronic hepatitis B and C in Saudi Arabia was estimated to be 2.72 % and 3.43 %, respectively, and hepatitis B and C related deaths were 2.2 and 6.61 deaths per 100,000 population, respectively (“CGHE,” 2020). Furthermore, the MOH reported that the incidence rates of hepatitis A, B and C in 2021 were estimated to be 0.23, 14.53, and 5.75 per 100,000 population, respectively (“MOH,” 2021a). Chronic viral hepatitis can lead to a multitude of complications including, liver cirrhosis, hepatocellular carcinoma and the need for liver transplant in Saudi Arabia (Altraif, 2018). This highlights the burden of these viral infections on the Saudi population and the need for measures and initiatives to eliminate them. The implementation of hepatitis B screening and vaccination programs that are mandated by the MOH and the introduction of new direct-acting antiviral therapy targeting hepatitis C in the past years have led to a decline in the prevalence of both hepatitis B and C in Saudi Arabia (Aljumah et al., 2019, Altraif, 2018).

### 2.1 Hepatitis A

Hepatitis A virus (HAV) is one of the greatest concerns in food safety that is endemic and prevalent in developing countries in contrast to developed countries, which commonly spread among children (Di Cola et al., 2021). HAV belongs to the family *Picornaviridae*, and it is transmitted mainly through the Fecal-oral route, contaminated sewage, and unprotected sexual contact. Symptoms of the disease range from asymptomatic to fulminant hepatitis, which include fever, diarrhea, vomiting, dark excrement, and jaundice with an incubation period of 14–28 days (Table 1) (Webb et al., 2020). It is a small single-stranded RNA (ssRNA) genome virus, measuring 27–32 nm in diameter and about 7.5 kb in length (Fig. 1). It has two infectious forms; the first is a non-enveloped HAV virion that passes out in the feces, and the second is a quasi-enveloped HAV virion (eHAV) that is secreted from infected cells (Randazzo and Sánchez, 2020). According to a recent study conducted by You Li *et al*. on Ifnar1−/− mice, which lack type-I interferon receptor, it confirmed the ability of RG7834 to completely block HAV infection, reduce both serum alanine aminotransferase activities and hepatocyte apoptosis. Despite numerous excellent vaccines, hepatitis outbreaks remain because there is no effective HAV therapy (Li et al., 2022). In 2008, Saudi Arabia included the hepatitis A vaccine for children within the universal mass vaccination program, which is given in two doses at 18 and 24 months of age (Table 2). Three vaccines are licensed in Saudi Arabia, an inactivated hepatitis A vaccine, two of which are available in the universal mass vaccination program, i.e., the Avaxim (Sanofi Pasteur – France) and Healive (Sinovac Biotech Ltd – China) vaccines (Badur et al., 2021).

**Table 1:** Summarization of the main characteristics of infectious viral diseases, their causative virus, modes of transmission, incubation period, and accompanying symptoms, and the total cases reported from 2018 to 2021 in KSA.*

Viral Diseases	Causative Virus	Transmission	Incubation period	Symptoms	Reported cases in KSA (2018–2021)*
Hepatitis A	Hepatitis A virus	Fecal-oral route, contaminated sewage, and unprotected sexual contact.	14–28 days	Fever, diarrhea, vomiting, dark excrement, and jaundice.	703
Hepatitis B	Hepatitis B virus	Vertical and horizontal transmission, exposure to infected blood, and unprotected sexual contact.	1–6 months	Often asymptomatic, jaundice, severe disease, may progress to liver failure.	23,219
Hepatitis C	Hepatitis C virus.	Exposure to infected blood.	4–20 weeks	Asymptomatic period, jaundice, weakness, gastrointestinal bleeding	10,543
Hepatitis D	Hepatitis D virus	Coinfection with hepatitis B virus	3–7 weeks	Asymptomatic period, ascites, muscle weakness, jaundice, dark urine, fatigue, malaise, and loss of appetite	19
Hepatitis E	Hepatitis E virus	Fecal-oral route, foodborne transmission, exposure to infected blood, and vertical transmission.	2–10 weeks	Jaundice, fatigue, loss of appetite, nausea, abdominal pain, fever, dark urine, and joint pain.	25
Dengue Fever	Dengue type 1, 2, 3, and 4 viruses	Mosquito-borne disease	2–7 days	Acute febrile, nausea, headache, vomiting, rash, pain behind the eyes, muscle and joint pain, bleeding and organ failure	10,986
Middle East Respiratory Syndrome	Middle East respiratory syndrome coronavirus	Contact with infected dromedary camels (direct or indirect), and human-to-human.	2–14 days	Pneumonitis, acute respiratory distress syndrome, respiratory failure, septic shock, and multi-organ failure.	483
COVID-19	Severe acute respiratory syndrome coronavirus 2	Contact with infected person (direct or indirect): through inhaling droplets, or touching contaminated surfaces and then touching the eyes, nose, and mouth.	1–14 days	Dyspnea, fever, cough, anosmia, headache, sore throat, sneezing, fatigue, and pneumonia.	841,469**
Influenza (Seasonal)	Influenza types A and B viruses	Contact with infected person (direct or indirect): through inhaling droplets, or touching contaminated surfaces and then touching the eyes, nose, and mouth.	1–4 days	Fever, Runny nose, Sore throat and cough, headache, fatigue, muscle pain.	18,541
Influenza Like Illness	Respiratory syncytial virus, Parainfluenza virus, adenovírus	Contact with infected person (direct or indirect): through inhaling droplets, or touching contaminated surfaces and then touching the eyes, nose, and mouth.	1–4 days	Fever, cough, and sore throat,	1424
Chickenpox	Varicella-zoster virus	Airborne transmission	10–21 days	Skin rash (which appears on the face, chest, and back, then spreads over the body parts) with small blisters causes itching, and fever.	10,523
Measles	Measles virus	Airborne transmission	10–14 days	Skin rash, otitis media, bronchopneumonia, laryngitis, tracheitis, and diarrhea.	99
Mumps	Mumps virus	Contact with the respiratory secretions of an infected person.	15–25 days	Fever, swelling of the parotid gland, pain, or asymptomatic infection.	1922
Rubella	Rubella virus	Contact with the respiratory secretions of an infected person, vertical transmission.	12–23 days	Red rash and fever.	594
Hand, Foot and Mouth Disease	Enterovirus A71, Coxsackie virus group A type 16	Fecal-oral route or by personal contact	3–7 days	Fever, vesiculation, and inappetence.	742
Conjunctivitis	Human adenovirus type 8, 3, and 37	Personal contact or shared infected items	2 days − 2 weeks	Conjunctival swelling, eye redness, pain and itching, photophobia, frequent tears accompanied by secretions.	381

* Statistics data were obtained from the Field Epidemiology Program (FETP) of MOH.

** Confirmed cases of COVID-19 disease in Saudi Arabia until April 2024 were obtained from (“Worldometers,” 2024).

**Table 2:** Mandatory vaccines in the Saudi schedule for childhood vaccinations. The data presented in this table was adapted from (“MOH,” 2018).

National Immunization Schedule												
Vaccine	Birth	2 Months	4 Months	6 Months	9 Months	12 Months	18 Months	24 Months	4–6 Years	11 Years	12 Years	18 Years
BCG	●			●								
HepB	●	●	●	●								
RV		●	●	●								
DTaP		●	●	●			●		●			
Hib		●	●	●			●					
PCV		●	●	●		●						
IPV		●	●	●								
OPV				●		●	●		●			
Measles					●							
MCV4					●	●						●
HepA							●	●				
Varicella							●	●	●			
HPV*										●	●	
MMR						●	●		●			
Tdap										●		

* For females only. BCG, Bacillus Calmette-Guerin; HepB, Hepatitis B; RV, Rotavirus; DTaP, Diphtheria, Tetanus, Pertussis; Hib, Haemophilus influenzae type b; PCV, pneumococcal conjugate vaccine; IPV, Inactivated poliovirus vaccine; OPV, oral polio vaccine; MCV4, Meningococcal conjugate vaccine; HepA, Hepatitis A; HPV, Human Papillomavirus; MMR, measles, mumps and rubella; Tdap, Tetanus, Diphtheria, Pertussis.

### 2.2 Hepatitis B

The prevalence rate of chronic hepatitis B virus (HBV) infection worldwide is estimated to be 257–291 million (Naggie and Lok, 2021). HBV is a member of the *Hepadnaviridae* family that has a partially double-stranded DNA genome (dsDNA) and it is small in size, about 3.2 kb in length (Fig. 1) (Tsukuda and Watashi, 2020). The main route of transmission is vertical transmission, which is from mother to newborn, or horizontal through exposure to infected blood or the reproductive system's secretions (Nguyen et al., 2020). The incubation period is variable and characterized by a long period ranging from 1 to 6 months. Clinical symptoms include jaundice and severe illness, although the disease is often asymptomatic, and may progress to liver failure (Table 1) (Zuckerman, 1996). HBV infection can lead to liver cirrhosis and hepatocellular carcinoma because it is non-cytopathic and capable of causing persistent infection of liver cells (Iannacone and Guidotti, 2022). HBV treatments are classified into two types of approved therapeutic agents which are direct-acting antiviral and immunotherapy. To achieve the endpoint, treatment requires inhibition of new hepatocyte infections, elimination of both hepatitis B surface antigen (HBsAg) and covalently closed circular DNA (cDNA), enhancement of immunity, and restoration of its function. HBV vaccine is applied for prevention; however, in chronically infected patients, the applied vaccine has failed to restore HBV-specific immunity which led to the production of new therapies (Alexopoulou et al., 2020). Most current therapies such as tenofovir or entecavir achieve only viral suppression, while rarely can remove surface antigens; thus, they must be given for lifelong. It is worth mentioning that there is a wide spectrum of antivirals under study that block the life cycle of the virus. There are several drugs in advanced stages of clinical development including bulevirtide, JNJ-6379, ABI-H0731, ARO-HBV, and REP-2139, in which expectations indicate that combination therapies of antivirals and immune modulators achieve the maximum benefits of treatment (Soriano et al., 2020). Saudi Arabia is one of the endemic countries with HBV infection in the Middle East (AlAteeq et al., 2022), since 1989, it has implemented a mandatory vaccination program for HBV immunization (Table 2), which has shown significant efficacy in declining cases over the past 20 years (Ahmad et al., 2022).

### 2.3 Hepatitis C

The Hepatitis C virus (HCV) represents a global health burden approximately 1 % of the world's population, is infected with this type of virus (Duncan et al., 2020). WHO has set the goal of eliminating hepatitis C and B by 2030 (Pedrana et al., 2020). Patients mostly are unaware of the presence of the infection due to the prolonged asymptomatic period (Rabaan et al., 2020). Healthcare and drug abusers are the main source of HCV (Hollande et al., 2020), which is primarily transmitted through the parenteral route contaminated with infectious blood (Schillie et al., 2020). The incubation period ranges from 4 to 20 weeks, and some symptoms and signs appear, including jaundice, weakness, and gastrointestinal bleeding (Table 1) (Hoofnagle, 1997). HCV belongs to the family *Flaviviridae* and the genus *Hepacivirus*. It is an enveloped, ssRNA virus (Fig. 1) (Rabaan et al., 2020). Several drugs are available in Europe, including sofosbuvir, velpatasvir, and voxilaprevir, either separately or combined in one tablet (Pawlotsky et al., 2020). In 2016, pan-genotypic direct-acting antivirals (DAA) were approved and recommended to treat HCV, which increased the effectiveness of treatment uptake (Shahid et al., 2021). Likewise, the treatment strategy followed in Saudi Arabia until the last few years was based on a combination of interferon and ribavirin. Since 2011, DAA has been approved in Saudi Arabia and led to higher sustained virologic response (Alarfaj et al., 2022). Egypt topped the world in the prevalence of hepatitis C, despite that, it has achieved great success in eliminating the disease by introducing sofosbuvir-based therapies into the national treatment program, as it was able to cure more than 4 million patients with hepatitis C (Waked, 2022, Waked et al., 2020). This significant progress can be implemented with suitable modification as a recommendation to help, reduce and control HCV disease regression and prevalence in Saudi Arabia. However, up to date, there is no effective vaccine to prevent HCV due to its characteristics, including about 10^−5^ mutations and high replication rates (Echeverría et al., 2021).

### 2.4 Hepatitis D

Although the hepatitis D virus (HDV) was previously thought to be local in Italy and southern Europe, it is now recognized as a global health problem, with an estimated 12 million patients in 2020 (Rizzetto et al., 2021). Furthermore, a lack of clinical epidemiological research has contributed to an incomplete understanding of the disease burden and, thus, a lack of effective treatments (Kumar, 2020). Based on the literature published over the past 40 years, many epidemiological surveys have attempted to estimate the number of hepatitis D-infected patients, but the actual number has not been accurately estimated. Different numbers indicate the heterogeneity of reports due to disparate recruitment methodologies and a lack of sufficient data (Rizzetto et al., 2021). HDV belongs to the family *Deltaviridae* and genus *Deltavirus*, which is a circular ssRNA genome (Fig. 1), enveloped, with a length of 1700 nt and a diameter of 36–43 nm (Wang and Tsai, 2021). HDV is an important contributor to HBV-associated liver illness, as it is estimated that one out of every 22 people infected with hepatitis B is also infected with hepatitis D (Stockdale et al., 2020). The HDV depends on the HBV for propagation; it cannot establish an infection on its own because it is a defective virus (Alfaiate et al., 2020). Chronic hepatitis D rapidly leads to dysfunction of the liver, cirrhosis and hepatocellular carcinoma (Caviglia and Rizzetto, 2020). Patients may be asymptomatic or present symptoms such as ascites, muscle weakness, jaundice, dark urine, fatigue, malaise, and loss of appetite, with an incubation period of 3–7 weeks (Table 1) (Farci and Niro, 2012). Previously, treatments were limited to pegylated interferon alfa until conditional approval was issued by the European Medicines Agency (EMA) for bulevirtide in 2020 (Sandmann and Cornberg, 2021). There are three new drugs under clinical trials, which include HBsAg secretion inhibitor (REP-2139), farnesyl-transferase inhibitor (Lonafarnib, LNF) and entry inhibitor (Bulevirtide, BLV; formerly Myrcludex B) (Lok et al., 2021).

### 2.5 Hepatitis E

More than three million cases of jaundice annually are attributed to the hepatitis E virus (HEV) and the fatality risk rate is high for pregnant women reaching 65 % (Azman et al., 2021). HEV belongs to the family *Hepeviridae* and the genus *Orthohepevirus*. The genetic material of the virus is a positive-sense ssRNA, linear, and non-enveloped, with a 27–34 nm diameter and an icosahedral capsid (Fig. 1) (Kar and Karna, 2020). HEV infection is transmitted through a foodborne route, and it is considered zoonotic. There are three possible ways of transmission, which include wastewater and human feces, infected workers in the field of food preparation, and consumption of infected animal meat. There are also other routes of transmission such as blood transfusions and vertical transmission (Di Cola et al., 2021). The incubation period ranges from 2 to 10 weeks, with clinical features such as jaundice, fatigue, loss of appetite, nausea, abdominal pain, fever, dark urine, and joint pain (Aggarwal and Krawczynski, 2000). In acute HEV infection, patients are usually asymptomatic, but they may develop icteric hepatitis in some cases (Table 1). Immunocompetent patients do not need specific treatment because of their ability to recover from infection. HEV infection in any case could cause chronic diseases and liver failure, particularly in pregnant women and older people (Aslan and Balaban, 2020). Patients are treated with broad-spectrum antiviral drugs, as there are no currently approved drugs. Drugs such as sofosbuvir, ribavirin and Peg-interferon 2 alpha are used in combination with each other to inhibit or reduce viral replication (Velavan et al., 2021).

## 3 Dengue fever (DF)

Viral Hemorrhagic Fever (VHF) are disease caused by several viruses that can transmit from infected animals, or insects, which are known as vector-borne diseases (VBDs) (“CDC,” 2023). Globally, VBDs account for more than 17 % of all infectious diseases and more than 700,000 deaths annually (“WHO,” 2020c). Epidemiological studies on the prevalence of VBDs in Saudi Arabia are not well established but have mainly targeted specific VBDs, such as DF and leishmaniasis, which are commonly found in specific regions of the country (Melebari et al., 2021, Abuzaid et al., 2020).

DF is a mosquito-borne disease caused by dengue viruses (DENVs) that form the dengue complex in the genus *Flavivirus*, family *Flaviviridae*. DENVs consist of four antigenically related serotypes, which are DENV-1, DENV-2, DENV-3, and DENV-4. The different variants are composed of ssRNA (Fig. 1), which are indistinguishable clinically. These DENVs can cause a different illness varying from non-symptomatic dengue infection to DF, dengue hemorrhagic fever (DHF), and dengue shock syndrome (DSS). DENVs are transmitted by four types of female mosquitoes, which are *Aedes aegypti, Aedes albopictus, Aedes scutellaris,* and *Aedes polynesiensis* (Chen et al., 2021, Sabir et al., 2021, Murugesan and Manoharan, 2020). However, the most common species in Saudi Arabia are *Aedes aegypti, and Aedes albopictus* (Alhaeli et al., 2016).

DF poses a global threat owing to its high mortality rates. It is a major public health concern that occurs in tropical and subtropical areas. The Centers for Disease Control and Prevention (CDC) reported that the infection causes fever (acute febrile) followed by nausea, vomiting, rash, aches and pains, which lasts typically 2–7 days. Pains may include eye pain located behind the eyes, muscles, joints, or bone pain. DF clinical features also include bleeding and organ failure (Table 1) (Chen et al., 2021, Dengue Symptoms and Treatment | CDC [WWW Document], 2021). Based on the (“PAHO,” 2022), there are around 2,812,155 cases in the Americas region and about 2,371,728 cases in the Southern Cone reported in 2022. In Saudi Arabia, a cross-sectional study on the incidence of DF transmission in Makkah between 2017 and 2019 found that the incidence rate among Saudi males was 23.54, 12.70, and 40.16 cases per 100,000 population for the years 2017, 2018, and 2019, respectively (Melebari et al., 2021). In 2021, the MOH reported that the incidence rate of DF was 10.03 cases per 100,000 population (“MOH,” 2021a).

To date, there is no effective medicine for DF treatment, which led to an urge to develop an efficient vaccine to prevent the progression of this disease. Several forms of DENV infectious vaccines have been developed that consist of live attenuated virus, live-attenuated chimeric recombinant virus, live-attenuated recombinant virus, recombinant protein, inactivated virus, and mRNA vaccines. Several licensed vaccines are used in several nations. The most known one is called (Dengvaxia® – France) sponsored by Sanofi which is a live-attenuated virus vaccine (Sabir et al., 2021). It is the only commercialized vaccine that exists; however, it cannot be administered to children under 9 years old who had a previous DF. Developing a successful dengue vaccine will be a potential need due to the recent significant high incidence rates worldwide (Park et al., 2022, Wilder-Smith, 2022).

A promising method for controlling DF is an intracellular insect bacterium called *Wolbachia*, which can rapidly spread into uninfected host mosquito populations by inducing cytoplasmic incompatibility in uninfected mosquitoes (Wilder-Smith, 2022). The *Wolbachia*-based biocontrol method has the potential to prevent and control dengue and other VBDs spread by *Aedes aegypti* mosquitoes (Cardona-Salgado et al., 2020). *Wolbachia*-based technology is a natural and environmentally friendly method that does not involve the use of chemicals or genetic modification of mosquitoes (Ross et al., 2023). The essence of *Wolbachia*-based biocontrol is to achieve so-called “population replacement”, i.e., the gradual eradication of wild mosquitoes and their replacement with *Wolbachia*-carrying mosquitoes with drastically reduced vector competence (Cardona-Salgado et al., 2020). This strategy has been lauded by the WHO and the CDC of the United States as one of the most effective and efficient methods for controlling DF (Mosquitoes with Wolbachia | CDC [WWW Document], 2022, Dengue control: three-year Indonesia trial shows promising results [WWW Document], 2020). Three major *Wolbachia* strains, wMel, wAlbB, and wMelPop, are currently undergoing prevention and control trials for arboviral infections (Ross et al., 2023).

In the Western province of Saudi Arabia where DF is endemic, *a Wolbachia*-based biocontrol strategy was implemented in 2021 proposing an alternative strategy for reducing DF. In a recent study, two Saudi *Wolbachia* strains (wAlbB and wMelM) were introduced into *Aedes aegypti* from Saudi Arabia for the release program that went in 2021 in Jeddah, a coastal city that has extreme weather and is ongoing (Pagendam et al., 2022, Ross et al., 2023). The study found that *Wolbachia* was able to reduce the spread of dengue virus type 2 (DENV2) in the Saudi Arabian mosquitoes, exhibited a complete maternal transmission, and exhibited cytoplasmic incompatibility (Ross et al., 2023). The *Wolbachia* also reduced the heat tolerance and egg viability of mosquitoes, with the *Wolbachia* strains exhibiting variable thermal stability. In addition, the study discovered indications of local adaptation, with Saudi Arabian mosquitoes exhibiting lower egg viability but higher adult desiccation tolerance compared to reference mosquitoes (Ross et al., 2023). These findings suggest that genetic background effects will influence *Wolbachia* invasion dynamics, reinforcing the need to use local genotypes in mosquito release programs, especially in extreme environments such as Jeddah (Ross et al., 2023).

Dengue Hemorrhagic Fever (DHF) is a life-threatening complication that needs a rapid response to manage and improve the clinical prognosis. It is a critical second phase of DF, which occurs when the fever drops to an approximate normal level for 24–48 h. DHF clinical symptoms are detected after 3–5 days of the fever that include plasma leakage, pleural effusion, bleeding, rise in hematocrit levels, thrombocytopenia with <100,000 platelets/μL, restlessness, abdominal pain, vomiting and sudden drop in temperature (Wang et al., 2020, Schaefer et al., 2023). DHF occurrence depends on several hypotheses that include cytokine storm, variation of the lipid profile, immunological enhancement, changes in viral virulence and genetic susceptibility. Some DHF patients were not infected with DENV but were infected with at least two different serotypes. Hemorrhage in patients infected with dengue is caused by different phenomena; for instance, coagulopathy (impaired coagulation), thrombocytopenia (abnormally low levels of platelets) and disruption in the epithelial cells as well as disseminated intravascular coagulation. There is an association between secondary dengue infection and severe dengue (Tsheten et al., 2021). DF patients with warning signs must be transferred to the hospital for intravenous fluid therapy administration and close monitoring to help reduce the chance of patients progressing to severe dengue and death (Tsheten et al., 2021). The mortality rate of untreated dengue severe fever is 10 %–20 %, which can be reduced to 1 % by appropriate supportive care (Schaefer et al., 2023).

## 4 Respiratory viral infections

Respiratory viruses are the main cause of lower respiratory tract infections (LRTIs), accounting for more than 60 % of all LRTIs that contribute to millions of deaths per year (Walker et al., 1994, Farrag et al., 2019). In Saudi Arabia, respiratory viral infections are estimated to affect around 15 % of the population per year (Farrag et al., 2019). Given the high number of foreign workers living in the country and the millions of Muslims traveling in and out of the country during the Hajj and Umrah seasons, the circulating respiratory viral patterns in Saudi Arabia are unique and can lead to a variety of viral outbreaks. The leading cause of viral respiratory diseases in the pediatric population in Saudi Arabia is Respiratory syncytial virus (RSV), with a prevalence of 23.5 % of pediatric patients with LRTIs (Alharbi et al., 2021). Among adults in Saudi Arabia, influenza virus is the most common viral respiratory disease, especially during Hajj season, with an overall prevalence of 5.9 % for influenza type A and 3.6 % for influenza type B (Safarpour et al., 2022). In 2022 the WHO reported that since the Middle East Respiratory Virus outbreak in 2012, the total number of cases was 2600 in 27 countries, with the majority of cases reported from Saudi Arabia (84 %, n = 2193) (“WHO,” 2022c). This highlights the magnitude of the health burden of respiratory viral infections in Saudi Arabia and their impact on the morbidity and mortality of the population. To minimize the impact of influenza on the Saudi population, the MOH has initiated Influenza Surveillance in Saudi Arabia that aims to establish and monitor trends of the virus, signal the start of the influenza season, provide data on the impact of influenza on the population, and plan appropriate control and intervention measures. In addition, Saudi Arabia’s response to the coronavirus pandemic was strategic and concordant with the WHO national response measures and health preparedness response pillars (A. Khan et al., 2021a).

### 4.1 Middle East Respiratory Syndrome (MERS)

Middle East Respiratory Syndrome (MERS) is an infection caused by the MERS coronavirus (MERS-CoV) that was initially isolated from a lung sample of a patient who suffered from severe pneumonia and died because of multiple organ failure at a hospital in Jeddah, Saudi Arabia in 2012. Nevertheless, in Jordan, in 2012, the MERS-CoV outbreak occurred, which led to the identification of MERS-CoV as a zoonotic pathogen transmitted from dromedary camels to humans. The findings showed that MERS-CoV, which is a large ssRNA virus (Fig. 1), can cause a lethal and fatal disease in humans (Memish et al., 2020). According to the WHO reports, MERS-CoV infection caused 851 deaths out of 2468 reported cases worldwide. The majority of cases were in Saudi Arabia which has the highest mortality rate, with approximately 36 %, of the reported cases (“WHO,” 2022a). However, the mortality rate decreased over the years starting from 45 % in 2014 to 41 % in 2015. A recent study has shown that the mortality rate reached 30 % in 2017 and 2018 (Al-Rabiaah et al., 2020). Until 11 March 2021, the total number of death cases was 886 (Ahmad, 2022). As mentioned, the infection can be transmitted from camels, which is a host reservoir specific for MERS-CoV, to humans, and also, human to human. However, the origin of the primary human infection remains unknown. The incubation period of MERS-CoV infection ranges from 2 to 14 days (about 2 weeks). The clinical features and symptoms vary from asymptomatic (mild upper respiratory illness) to rapidly progressive pneumonitis, acute respiratory distress syndrome, respiratory failure, septic shock, and multi-organ failure (Table 1). Approximately 25 %–50 % of the infected cases were reported as asymptomatic-to-mild infection (Memish et al., 2020). To date, there are no specific treatments or vaccines available for MERS-CoV that have been endemic for more than 10 years in Saudi Arabia according to the WHO. Nevertheless, a recent study on post-MERS-CoV infection in recovered individuals in Saudi Arabia has shown cellular immune responses which last for 6–9 years (Alhabbab et al., 2023, Middle East respiratory syndrome coronavirus (MERS-CoV) [WWW Document], 2022b).

### 4.2 Coronavirus Disease 2019 (SARS-COV-2)

The novel coronavirus 2019 caused by the SARS coronavirus-2 (SARS-CoV-2) named by the Coronavirus Study Group, is originated in bats and transmitted to humans through unknown intermediary animals in December 2019 in Wuhan, Hubei Province, China (Singhal, 2020, Krishnan et al., 2021). SARS-CoV-2 is an RNA virus (Fig. 1), which belongs to the genus *betacoronavirus* (Alexandersen et al., 2020, Sadeghi Dousari et al., 2020). SARS-COV-2 infection caused more than 6 million deaths around the world in a few years, which had a critical impact on humanity (Simonsen and Viboud, 2021). Most recently, the coronavirus pandemic was responsible for millions of deaths globally, and as of January 30th, 2024, the number of confirmed cases in Saudi Arabia is 841,469 while the mortality was 9,646 (“Worldometers,” 2024). SARS-CoV-2 is spread through contact with an infected person (direct or indirect): inhaling droplets, or touching contaminated surfaces and then touching the eyes, nose, and mouth (Khan et al., 2021b). Symptoms range from mild to severe and include dyspnea, fever, cough, anosmia, headache, sore throat, sneezing, fatigue, and pneumonia (Singh et al., 2021), with an incubation period of 1–14 days (Zhang et al., 2021). The infection has a high prevalence rate internationally which led to quarantine and high restrictions to control and reduce the infection rate globally (Ferreira et al., 2021). This infection affected human life, the environment, economics, and transportation in many countries (Nundy et al., 2021).

Different types of vaccines were rapidly developed to prevent and control the infection rate (Al-Amer et al., 2022). One hundred eighty-four SARS-COV-2 vaccines were developed and only 105 vaccines were used in clinical for approval, eighteen of which were approved that included whole virus inactivated, live attenuated, viral vector, protein-based and nucleic acid vaccines (Ndwandwe and Wiysonge, 2021). In Saudi Arabia, four vaccines were approved and used to prevent SARS-COV-2 infection which are Pfizer-BioNTech (United States), Moderna (United States), Oxford-AstraZeneca (United Kingdom) and Janssen (United States) (Ministry of Health Saudi Arabia [WWW Document], 2023, Alhazmi et al., 2021). Pfizer-BioNTech and Moderna vaccines have used a new technique, i.e., the mRNA-based vaccination, which provided significant immunity and prevented the transmission of SARS-COV-2 infection (Dighriri et al., 2022). Oxford-AstraZeneca vaccine is a recombinant vaccine ChAdOx1 SARS-COV-2 that was produced by utilizing replication-deficient chimpanzee adenovirus to obtain spiked antigen protein of SARS-CoV-2 (Onyeaka et al., 2021). The Janssen vaccine production uses an existing technology with modified adenovirus, which is a common cause of respiratory infections that produces a key part of SARS-CoV-2 DNA used as a vaccine (Livingston et al., 2021). All vaccines were reported as effective among individuals in the clinical risk group by taking more than one administrative dose (Whitaker et al., 2022, Kaura et al., 2022). However, there were about 43 % of the public in the Gulf Cooperation Council (GCC) countries had vaccine hesitance issues (Alsalloum et al., 2022). For example, in Saudi Arabia, parents had concerns for their children to be vaccinated against SARS-CoV-2, which includes safety, efficacy and side effects (Sayed, 2024). Despite the availability of some anti-SARS-COV-2 antivirals that have shown an impact against SARS-CoV-2, the Saudi Authorities have made tremendous efforts to promote the confidence and the administration of SARS-CoV-2 vaccines through social marketing and awareness and vaccination campaigns for all age groups. These efforts have led to a high percentage of public vaccination against SARS-COV-2, reducing the need for antiviral medications generally (Alhraiwil et al., 2024).

### 4.3 Influenza (Seasonal)

The influenza seasonal infection is an annual prevalent that causes a high rate of morbidity and mortality, which range from 290,000 to 650,000 mortalities worldwide (Tyrrell et al., 2021, Dunning et al., 2020). RNA viruses are the main cause of influenza that belong to the *Orthomyxoviridae* family (Fig. 1), which can be classified based on their antigenic differences into four types; i.e., influenza A to D (Zhu et al., 2023). Influenza A and B cause seasonal influenza which use the shift and drift mechanisms to change their antigenic properties to evade the immune system and cause the infection more than once (Tyrrell et al., 2021, Wang et al., 2022). Transmission occurs when inhaling the droplets of an infected person, or touching contaminated surfaces and then touching the face, and is characterized by fever, Runny nose, Sore throat and cough, headache, fatigue, and muscle pain, with an incubation period of 1–4 days (Table 1) (“WHO,” 2023). The best approach to prevent influenza infection is vaccination (Rockman et al., 2020); however, the seasonal influenza vaccination is annually reformulated and should be acquired for effective prevention (Nuwarda et al., 2021). In 1938, the first monovalent virus vaccine for influenza A and bivalent vaccine for influenza B were developed, which were generated in embryonated hens' eggs using live attenuated or inactivated viruses' formulations. There are several technologies for developing seasonal influenza vaccines including cell culture vaccines, recombinant proteins, nucleic acid vaccination technologies, viral vectors (Rockman et al., 2020), egg-based vaccines, and attenuated vaccines (Nypaver et al., 2021). Influenza vaccination is essential for protecting the most vulnerable individuals and exposed individuals such as healthcare workers and it is considered mandatory for healthcare workers in Saudi Arabia (Tanner et al., 2021). The seasonal influenza infection rate has noted a large reduction since the SARS-COV-2 pandemic, mostly due to the precautionary and restriction measures that have been applied (Dhanasekaran et al., 2022).

### 4.4 Influenza Like Illness (ILI)

ILI infection is a group of symptoms that occur similarly to influenza, such as fever, cough, and sore throat, which is caused by different respiratory viruses including rhinovirus, adenovirus, human RSV, and parainfluenza virus (PIV) (Spencer et al., 2022, Pellegrinelli et al., 2022). However, the most common virus that causes ILI is RSV which often occurs in children (Kondratiuk et al., 2021, Faye et al., 2022). ILI is commonly spread through contact with an infected person (direct or indirect) (Fauzi et al., 2023), with an incubation period of 1–4 days (Table 1) (Stellrecht, 2017). In Saudi Arabia the number of cases infected with ILI is about 1424 from 2018 to 2021 (MOH, 2021b), however, the number of cases in the United States is 9 million to 49 million annually (Spencer et al., 2022, Jones et al., 2021), (Blanchet Zumofen et al., 2023), which is considered a leading factor for high mortality and morbidity (Mezlini et al., 2022). ILI prevention and controlling approaches are vaccination and antiviral medication for treatment (Blanchet Zumofen et al., 2023); yet, vaccination plays a critical role in reducing the prevalence rate internationally and reducing the severity of the infection (Khan et al., 2021c, Kaaijk et al., 2022). Influenza vaccines can be utilized for both infections, influenza and ILI.

## 5 Exanthematous viral infections

Exanthems are mainly caused by viral infections, including varicella, measles, mumps, rubella, enterovirus, and many other viruses and bacteria, but can also be due to noninfectious etiologies (Keighley et al., 2015). Exposure of the mucosal tissue to such viruses, or toxins produced by microorganisms, leads to an immune reaction that manifests as skin rashes with diverse characteristics that can be localized or generalized (Ting and Nixon, 2021). In Saudi Arabia, exanthematous viral infections often occur among children. One prospective study in King Abdulaziz Medical City in Riyadh reported that 78 % (2984/3802 cases) of chickenpox also known as varicella-zoster virus (VZV) cases were among children less than 15 years of age (Almuneef et al., 2006). In addition, according to the WHO/UNICEF Joint Reporting Form on Immunization (JRF), the incidence rates of measles, mumps, and rubella in Saudi Arabia in the year 2022 were 6.8 and 2.8, and 2.3 per 1,000,000 total population, respectively (“WHO,” 2020b). Advancements in vaccines have played a major role in the significant decrease of exanthem infection cases globally and led to the eradication of multiple viral diseases; yet, the risk of outbreaks still exists (“CDC,” 2024). Such outbreaks can lead to substantial health and socioeconomic burdens on countries (Yin et al., 2021). Governmental policies, such as vaccine mandates, help in reducing and mitigating the risks of outbreaks and maintaining the overall health and well-being of the public.

### 5.1 Chickenpox

Chickenpox is a contagious viral infectious disease caused by the VZV (Ayoade and Kumar, 2023), which has a dsDNA genome (Fig. 1) (Irham et al., 2023). It is an airborne disease, and the infection begins in the mucous membrane of the upper respiratory tract, where it multiplies and then spreads to the lymphoid tissues (Yu et al., 2020). The main symptom of chickenpox is skin rash, which appears as small, itchy blisters on the chest, back, and face and spreads to other parts of the body while accompanied by a mild fever (Ayoade and Kumar, 2023). The incubation period ranges from 10 to 21 days (Table 1) (Arashova, 2023). It occurs most commonly in children, adolescents, and young adults (Khallafallah and Grose, 2022). VZV infection is estimated to be around 750,000 and 800,000 cases globally every year (Abdukhuhidovich et al., 2020), and some reports showed that the significant incidence rate in Latin America is estimated to be 270 cases per 100,000 people, which is considered a high prevalence rate. Therefore, the implementation of a vaccination program to prevent and control could be the ideal strategy to limit the spread of this disease (Barrenechea and Bastos, 2020). VZV is responsible for both chickenpox and shingles; hence, some communities tolerate chickenpox infection among children to reduce rates of shingles among the elderly, these policies are considered unethical due to their harm to children (Malm and Navin, 2020). In 1998, the WHO recommended immunization against the chickenpox virus with a live attenuated virus vaccine (Barrenechea and Bastos, 2020), such as (Varivax™ and Varilrix™ – United States), as a preventative measure to control this disease (Sile et al., 2022). Hence, it is included in the mandatory National immunization schedule available in Saudi Arabia (Table 2).

### 5.2 Measles

According to published reports, the measles virus (MeV) remains responsible for more than 100,000 child deaths annually around the globe (Griffin, 2020). In 2010, the WHO enacted three milestones for controlling measles by 2015: increasing vaccine coverage for children, reducing the annual incidence rate, and reducing mortality (Patel et al., 2020). This initiative has recommended national immunization programs which included two doses of the measles-containing vaccine MCV (Wilder-Smith and Qureshi, 2020). MeV is a member of the *Paramyxoviridae* family and *Morbillivirus* genus that has a negative-sense ssRNA (Fig. 1) (Ayasoufi and Pfaller, 2020), and a length of 15 kb long (Karamitros et al., 2021). Measles is an airborne virus and infection that can occur through respiratory droplets and aerosols (Laksono et al., 2020). The infection begins in the lower respiratory tract, and then progresses towards the upper respiratory, spreading to the skin and conjunctiva toward the lymphocytes, as the virus is characterized by its high affinity for these cells (Rabaan et al., 2022). The possibility of infection transmission from a contagious patient during the four days before and four days after the rash appears (Bianchi et al., 2021). The incubation period of the MeV ranges from 10 to 14 days (Rabaan et al., 2022), and the clinical signs appear within 9–19 days (Anichini et al., 2020). Represent the most common complications: otitis media, bronchopneumonia, laryngitis, tracheitis, and diarrhea are considered the most common complications of measles (Table 1) (Bianchi et al., 2021). People with measles develop lifelong immunity when they recover from it (Anichini et al., 2020). The prophylactic vaccine is the best approach to prevent this disease owing to the absence of any specific treatment against MeV (Anichini et al., 2020). Global mass vaccination has reduced the infection rate by 99.9 % worldwide and has proven the safety of measles-containing vaccines (Bianchi et al., 2021). Therefore, it is included in the mandatory National immunization schedule available in Saudi Arabia (Table 2).

### 5.3 Mumps

Despite vaccinations, reports have shown that many countries had outbreaks of the mumps virus (MuV) over the last two decades (Gokhale et al., 2023). The unusual outbreak of this disease in the United States occurred between 2016 and 2017, which raised questions about its extent and its relationship to the previous outbreaks (Wohl et al., 2020). Mumps is an acute respiratory disease that is contagious and mainly affects children (Su et al., 2020), and its outbreak is affected by regional and seasonal differences. Therefore, it can be controlled by exploring the impact of climatic factors on the prevalence of the infection (Zha et al., 2020). MuV is a negative-sense, ssRNA virus that belongs to the *Paramyxoviridae* family and the *Orthorubulavirus* genus (Fig. 1) (Ueo et al., 2020). Transmitted through personal contact and respiratory droplets, the virus incubation period might range from 15 to 25 days (Almansour, 2020). It is characterized by fever, swelling of the parotid gland, pain, or asymptomatic infection (Hahné et al., 2017) (Table 1). Since the reproduction of the virus begins in the nasopharynx and regional lymph nodes, a saliva sample can be used to detect MuV (Connell et al., 2020). It also affects the parotid gland, mammary glands, ovary, testis, pancreas, and kidney, among other organs. In some cases, mumps could cause meningitis and encephalitis by affecting the central nervous system (CNS) (Ueo et al., 2020). Mumps can be distinguished from other causes of parotitis using reverse transcription polymerase chain reaction (RT-PCR) and the detection of immunoglobulin M (IgM) antibodies (Lam et al., 2020). Since 1946, several mumps vaccines have been developed, and most of the Group of Twenty (G20) and European countries now include the trivalent measles, mumps, and rubella vaccine (i.e. MMR vaccine) in their childhood immunization programs (Almansour, 2020). Hence, it is included in the mandatory National immunization schedule available in Saudi Arabia (Table 2). One of the reasons that contributed to the outbreak of mumps among vaccinated individuals was the mismatch between the vaccine strain and the intensity of exposure, which led to discussions about new strategies to prevent future outbreaks and the decision to include a third dose of the MMR vaccine (Lam et al., 2020).

### 5.4 Rubella

Since 2012, the WHO has set goals to eliminate rubella, and by 2020, nearly half of the world's countries have very limited cases annually (Zimmerman et al., 2022). Rubella virus (RUBV) is a positive-strand RNA, which belongs to the *Matonaviridae* family (Fig. 1) and genus *Rubivirus* (Das and Kielian, 2021). This virus can cause an acute infection characterized by mild symptoms, such as rash and fever (TERRACCIANO et al., 2020). The incubation period ranges from 12 to 23 days (Best, 2007). Rubella is an airborne disease that infects children, adults, and pregnant women, and it is easily transmitted through breathing droplets or contact with respiratory secretions (Winter and Moss, 2022). When the infection occurs during the first trimester of pregnancy, it can cause fetal death or congenital rubella syndrome (CRS) (Table 1), which is known to affect four babies out of every one thousand live births (TERRACCIANO et al., 2020). This syndrome causes serious lifelong clinical symptoms including heart defects, cataracts, hearing impairments, jaundice, hepatosplenomegaly, and microcephaly (Winter and Moss, 2022), in addition to thyroid dysfunction and diabetes (TERRACCIANO et al., 2020). In 1969, rubella-containing vaccines (RCVs) were introduced in Europe and the United States, which played a vital role in reducing rubella and CRS infections (Motaze et al., 2021). The vaccination strategy primarily targets children 14 years or under, and the WHO recommended achieving a minimum immunization rate of at least 80 % with a single dose that can provide lifelong protection (Zimmerman et al., 2022). Therefore, it is included in the mandatory National immunization schedule available in Saudi Arabia (Table 2).

### 5.5 Hand, foot and mouth disease

Hand, foot, and mouth disease (HFMD) is a rising concern in the Asia-Pacific region due to recurrent cyclical outbreaks (Lim et al., 2020). HFMD viruses are ssRNAs belonging to the *Picornaviridae* family and *Enterovirus* genus (Fig. 1) (Zhao et al., 2020), which are mainly infected with enteroviruses 71 (EV71) and coxsackievirus-A16 (CoxA16) (Yi et al., 2022). Usually, children under five years are highly susceptible and the viruses can be transmitted through the fecal–oral route or by personal contact (Yi et al., 2022). HFMD causes mild symptoms, such as fever, vesiculation, and inappetence, and in some cases, it may progress to neurological complications that might lead to death (Jiang et al., 2021), with an incubation period of 3–7 days (Table 1) (Yang et al., 2017). In 2016, three inactive monovalent EV-A71 vaccines were licensed in China and Greece, which held a huge potential to change the epidemiological trend of HFMD disease and show efficacy (Wang et al., 2021). Another study reported that a multivalent HFMD vaccine should be considered to protect against HFMD (Fang and Liu, 2022). In Saudi Arabia, up to date, there are no approved vaccines or treatments for HFMD for humans. However, there are some National efforts to prevent HFMD spread by animal vaccination strategies and enhanced hygiene control awareness.

## 6 Viral conjunctivitis

All over the world, conjunctival cases abound in the ophthalmology clinic, and viral conjunctivitis cases are more common than bacterial conjunctivitis, while allergic conjunctivitis affects half of the world’s population (Azari and Arabi, 2020). Adenoviral conjunctivitis is a common viral infection that infects the ocular surface of the eye (Tabbara et al., 2010). Adenovirus is highly transmissible and can lead to outbreaks that can impact public health (Johnson et al., 2023). Studies on the prevalence of viral conjunctivitis in Saudi Arabia are limited. One study from a single center evaluated the serotypes of viral conjunctivitis from 65 patients and found that the most common causes of adenoviral keratoconjunctivitis in Saudi Arabia were adenovirus types 8, 3, and 37 (Tabbara et al., 2010). Further epidemiological studies on the prevalence of viral conjunctivitis in Saudi Arabia are needed to assess the status of viral conjunctivitis in Saudi Arabia, which may aid in the development of preventative and management guidelines. The term conjunctivitis refers to a group of diseases associated with the inflammation of the conjunctiva. Viral infections are responsible for 80 % of the cases, while Human adenoviruses (HAdVs) represent 65–90 % of the cases. Other viruses such as herpes simplex virus (HSV), VZV, and *Molluscum contagiosum* virus are all associated with conjunctivitis, but to a lesser extent (Yeu and Hauswirth, 2020). Adenovirus belongs to the family *Adenoviridae* and the genus *Mastadenovirus*, which is a non-enveloped virus with icosahedral capsids consisting of a linear dsDNA genome (Fig. 1) (Labib et al., 2020). It is worth noting that this virus has multiple modes of transmission, whether through personal contact or shared infected items and hence, contributes to the epidemiology of the infection. Therefore, it is necessary to raise public awareness to limit its spread (Azari and Arabi, 2020). Conjunctivitis is clinically characterized by conjunctival swelling, eye redness, pain and itching, photophobia, and recurrent tears (Muto et al., 2023), With an incubation period ranging from 2 days to 2 weeks (Table 1) (Kimura et al., 2009). Antivirals are often used in the treatment plan, such as ganciclovir, ribavirin, and cidofovir owing to their potential therapeutic benefits; yet, there is no Food and Drug Administration (FDA) approved antiviral treatment for conjunctivitis (Labib et al., 2020).

## 7 Viral infections management strategies in Saudi Arabia

In Saudi Arabia, there are specific strategies that are followed to control and reduce viral infections. For healthy life quality control, there are childhood vaccination programs, an awareness platform of MOH, and mandatory guidelines for infectious disease prevention during Hajj season.

Childhood vaccination programs start from birth to the age of 18 years and are maintained and controlled based on the national immunization schedule (Table 2). One of the MOH efforts was establishing a reminder service for children's health preservation and protection from diseases. This service reminds parents about the basic vaccinations, which are against the targeted diseases for immunity according to the MOH schedule, dates through electronic mail or mobile phone a week before the date of their child's vaccination (“GOV.SA,” 2024).

The MOH has provided and issued a list of health requirements, recommendations, and prevention for Pilgrims to Saudi Arabia for Hajj. However, pilgrims within the Kingdom have also required several vaccines. Before applying for Hajj, pilgrims from other countries are required to be vaccinated against several diseases, which are meningococcal meningitis, poliomyelitis, and yellow fever. Moreover, they are recommended to be vaccinated against SARS-CoV-2, and seasonal influenza (Table 3). Nevertheless, the guidelines for pilgrims are one of the MOH efforts that can be easily accessed through the MOH website (“MOH,” 2022).

**Table 3:** Pilgrims Vaccinations for both citizens and travelers. The data presented in this table was adapted from (“MOH,” 2022).

Vaccinations for Citizens		
Disease		Approved Vaccine
SARS-COV-2		Pfizer-BioNTech, Moderna, Novavax.
Meningococcal meningitis		Meningitis quadrivalent vaccine
Seasonal influenza		Seasonal Influenza vaccine.

Vaccinations for Travelers		

	**Disease**	**Approved Vaccine**

Required vaccinations	Meningococcal meningitis	Quadrivalent Polysaccharide Vaccine, Quadrivalent Conjugated.
	Poliomyelitis*	Bivalent oral polio vaccine (bOPV), or inactivated polio vaccine (IPV), or oral polio vaccine (OPV).
	Yellow Fever*	Yellow Fever vaccine.
Recommended vaccinations	SARS-COV-2	Pfizer-BioNTech, Moderna, Oxford-Astrazeneca, Janssen.
	Poliomyelitis*	Inactivated polio vaccine (IPV), or oral polio vaccine (OPV).
	Seasonal Influenza	Seasonal Influenza vaccine.

* For specific countries.

## 8 Conclusion

The emergence of viral infectious diseases continues to threaten global public health. Viruses have always been associated with infectious disease outbreaks due to their wide range of species, hosted in different vectors, ease of transmission to humans, and increased rates of infectivity. Rapid detection of viral disease trends and patterns would maintain population health and facilitate disease elimination. It is essential to understand the epidemiology of viruses, transmission patterns, and the factors underlying the emergence of the disease. Saudi Arabia’s MOH is the responsible entity for public health surveillance and awareness in the Kingdom, which aims to control and prevent the spread of diseases including viral infections. The reported viral diseases in Saudi Arabia based on the MOH include hepatitis diseases (A–E), DF, respiratory viral diseases (MERS, SARS CoV-2, seasonal influenza, and influenza-like illness), exanthematous viral diseases (chickenpox, measles, mumps, rubella, and hand, foot, and mouth disease), and conjunctivitis. Strengthening and standardizing public health surveillance is critical to identifying indicators of the underlying causes of emerging viral diseases in the Kingdom. The rise of the Public Health Authority in Saudi Arabia has served the Kingdom’s community through monitoring, measuring, evaluating, controlling, and preventing all risks that could threaten public health, including communicable and noncommunicable diseases, and other health challenges, which can play a vital role in combating emerging and reemerging viral diseases in Saudi Arabia. Moreover, the availability of effective vaccines and antivirals ease the management of viral disease outbreaks. Other disease control strategies such as improved hygiene and sanitation facilities, eliminating arthropod vectors, and quarantine can be considered effective approaches to limit the spread of viral infections. Therefore, increasing the research and development efforts in developing vaccines, antiviral therapies, and other viral disease outbreak programs is an essential need to enhance the preparedness and response levels of countries against any future viral disease epidemics.

## Institutional review board statement

Not applicable.

## Informed consent statement

Not applicable.

## CRediT authorship contribution statement

**Noura M. Alshiban:** Investigation, Writing – original draft, Project administration. **Munirah S. Aleyiydi:** Investigation, Project administration, Writing – original draft. **Majed S. Nassar:** Investigation, Writing – original draft. **Nada K. Alhumaid:** Investigation, Writing – original draft. **Thamer A. Almangour:** Investigation, Writing – original draft. **Yahya M.K. Tawfik:** Investigation, Writing – original draft. **Laila A. Damiati:** Investigation, Visualization. **Abdulaziz S. Almutairi:** Resources, Supervision, Validation, Writing – review & editing. **Essam A. Tawfik:** Conceptualization, Supervision, Writing – review & editing.

## Declaration of competing interest

The authors declare that they have no known competing financial interests or personal relationships that could have appeared to influence the work reported in this paper.

## Data Availability

The authors confirm that the data supporting the findings of this study are available within the article.
